# Oral adverse events due to zinc deficiency after pancreaticoduodenectomy requiring continuous intravenous zinc supplementation: a case report and literature review

**DOI:** 10.1186/s12903-022-02088-3

**Published:** 2022-03-03

**Authors:** Hironobu Hata, Yojiro Ota, Katsuhiko Uesaka, Yutaka Yamazaki, Tsubasa Murata, Chika Murai, Kazuhito Yoshikawa, Kenji Imamachi, Takashi Yurikusa, Yoshimasa Kitagawa

**Affiliations:** 1 Division of Dentistry and Oral Surgery, Shizuoka Cancer Center Hospital, Shizuoka, Japan; 2 Department of Dentistry and Oral Surgery, National Hospital Organization Hokkaido Cancer Center Hospital, Sapporo, Japan; 3 Division of Hepato-Biliary-Pancreatic Surgery, Shizuoka Cancer Center Hospital, Shizuoka, Japan; 4 Gerodontology, Department of Oral Health Science, Faculty of Dental Medicine, Hokkaido University, Sapporo, Japan; 5 Department of Dental and Oral Surgery, Tomakomai City Hospital, Tomakomai, Japan; 6 Faculty of Dental Medicine, Oral Diagnosis and Medicine, Hokkaido University, Sapporo, Japan

**Keywords:** Zinc deficiency, Dysgeusia, Pancreaticoduodenectomy, Total parenteral nutrition, Case report

## Abstract

**Background:**

Zinc is mainly absorbed in the duodenum and proximal jejunum, which are removed during pancreaticoduodenectomy (PD). Little is known about the adverse oral events and skin disorders caused by zinc deficiency after PD. Herein, we reviewed studies on the development of zinc deficiency after PD and reported about a patient with zinc deficiency after PD who required home intravenous zinc replacement.

**Case presentation:**

A 73-year-old woman with glossitis, taste disorder, and acrodermatitis enteropathica-like eruption on her fingers presented to the Division of Dentistry and Oral Surgery 69 days after PD. Her serum zinc level markedly decreased to 30 μg/dL. Oral zinc administration was inadequate to treat hypozincemia after PD; therefore, multi-trace elements were injected intravenously during readmission. Her serum zinc levels recovered, and her lesions gradually improved. Furthermore, a central venous port was implanted to maintain normal serum zinc levels, and she continued self-injecting zinc at home.

**Conclusions:**

Zinc deficiency after PD rarely occurs. The clinical oncologist community, including dentists responsible for the oral care of cancer patients, should be aware of the oral adverse events, such as dysgeusia, glossitis, and oral pain, associated with zinc deficiency after cancer surgery and that induced by chemotherapy or head and neck radiation therapy.

## Background

Zinc is an essential trace element for humans, which, when deficient, can cause various pathological conditions, such as dermatitis, hair loss, anemia, dysgeusia, impaired development, gonadal dysfunction, and wound healing disorders [1, 2]. The role of zinc in taste functions is appreciable at various levels of body organization, such as taste buds and the taste sensation transmission [3]. Zinc deficiency secondary to any etiology leads to taste disturbances; thus, zinc depletion is corrected for patients presenting with taste imbalances [4–7]. It has been observed in vivo that zinc administration improves decreased taste bud cell proliferation caused by zinc deficiency [8]. Zinc administration improves taste in 50–82% of patients suffering from taste disorders [9]. Dysgeusia during cancer treatment is mainly reported in systemic chemotherapy and in surgery and radiation therapy for head and neck cancer [10]. However, there are a few reports of taste disorders secondary to zinc deficiency associated with surgery in other regions of the body.

Pancreaticoduodenectomy (PD) is the standard operation for periampullary cancers. Zinc is primarily absorbed in the duodenum and proximal jejunum, which are mostly resected during PD [11, 12] and may result in nutritional sequelae due to zinc deficiency [13]. Armstrong et al. reported that the incidence of zinc deficiency after PD is 50% [14]. Yu et al. [15] reported that 68% of the patients in their study had low serum zinc levels, and 43% exhibited clinical symptoms related to zinc deficiency following PD. However, the frequency of taste disorders was not described in either study. The incidence of zinc deficiency with acrodermatitis enteropathica after PD is approximately 0.3% in a high-volume hospital [13].

Little is known about the adverse oral events and skin disorders caused by zinc deficiency after PD. Therefore, we report a patient who experienced zinc deficiency with dysgeusia, glossitis, and acrodermatitis enteropathica-like eruption after PD.

## Case presentation

**Fig. 1:**
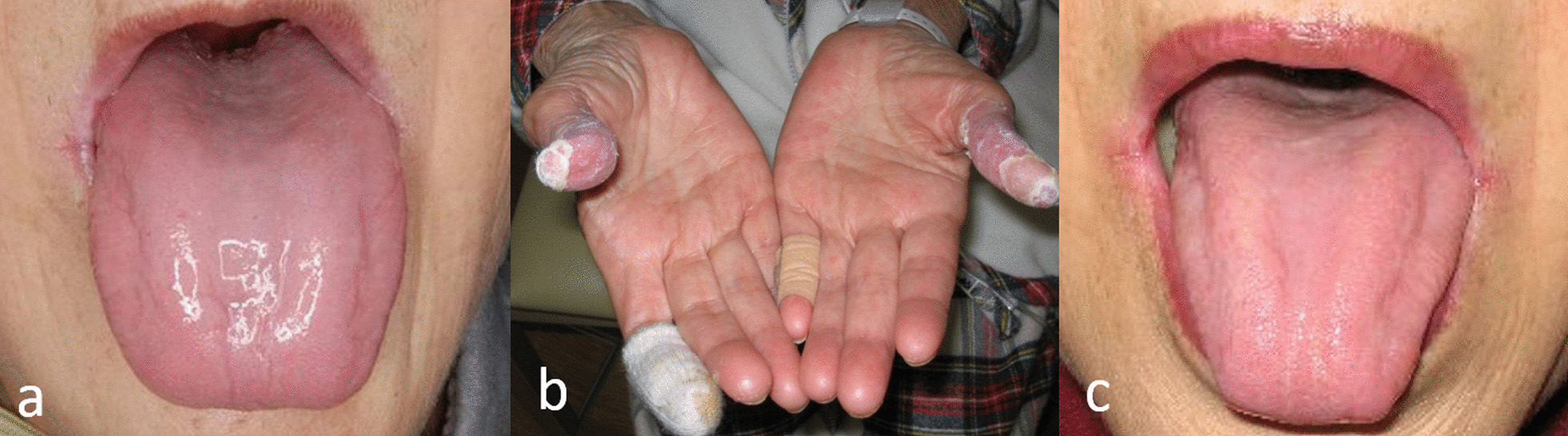
**a** Glossitis with atrophy of the lingual papillae and erythema. **b** Picture of the bilateral thumbs showing acrodermatitis enteropathica-like eruption and abnormal keratinization. **c** Picture showing improved atrophy of the lingual papillae as a result of proper zinc supplementation, but refractory angular cheilitis

A 73-year-old woman with pancreatic head adenocarcinoma underwent pancreatoduodenectomy (PD) at the Division of Hepato-Biliary-Pancreatic Surgery, Shizuoka Cancer Center Hospital, Shizuoka, Japan, in July 2005. The patient’s height, body weight, and body mass index were 154 cm, 56 kg, and 23.6 kg/m^2^, respectively. The patient was discharged 40 days postoperatively; however, approximately 1 month after discharge, she visited the hospital’s Division of Dentistry and Oral Surgery with a chief complaint of tongue pain with dysgeusia. The first examination revealed glossitis characterized by complete atrophy of the lingual papillae, which became erythematous; this is a symptom of glossitis (Fig. 1a). Furthermore, remarkable taste disorder (hypogeusia) and oral pain were reported. The angle of the mouth had stomatitis with erosive changes. The extremities showed acrodermatitis enteropathica-like eruption and abnormal keratinization (Fig. 1b). Blood test results showed hypoproteinemia and hypoalbuminemia (total protein (TP) 5.1 g/dL, albumin (ALB) 2.4 g/dL) (Table 1). Examination of trace elements showed remarkably lower serum zinc and copper levels (30 μg/dL and 40 μg/dL, respectively) (Table 1). We diagnosed malnutrition, dysgeusia, glossitis, angular cheilitis, and acrodermatitis enteropathica due to zinc deficiency. However, she had no obvious frequent diarrhea or steatorrhea.

**Table 1:** Data of blood tests at the time of initial examination

Parameter	Reference limits	Initial examination
Total protein	6.3–8.0 g/dL	5.1
Albumin	3.9–4.9 g/dL	2.4
White blood cell	3.3–8.2 10^3^/μL	7.03
Red blood cell	376–500 10^4^/μL	400.0
Hemoglobin	11.5–14.7 g/dL	12
Hematocrit	34.5–44.3%	36.2
Platelets	10^6^/μL	16.7
Aspartate aminotransferase	7–38 IU/L	45
Alanine aminotransferase	4–44 IU/L	46
Alkaline phosphatase	100–330 IU/L	368
Carcinoembryonic antigen	< 5.0 ng/mL	18
Colorectal carcinoma antigen 19-9	< 37.0 U/mL	15
Zinc	80–140 μg/dL	30
Copper	60–130 μg/dL	40

**Fig. 2:**
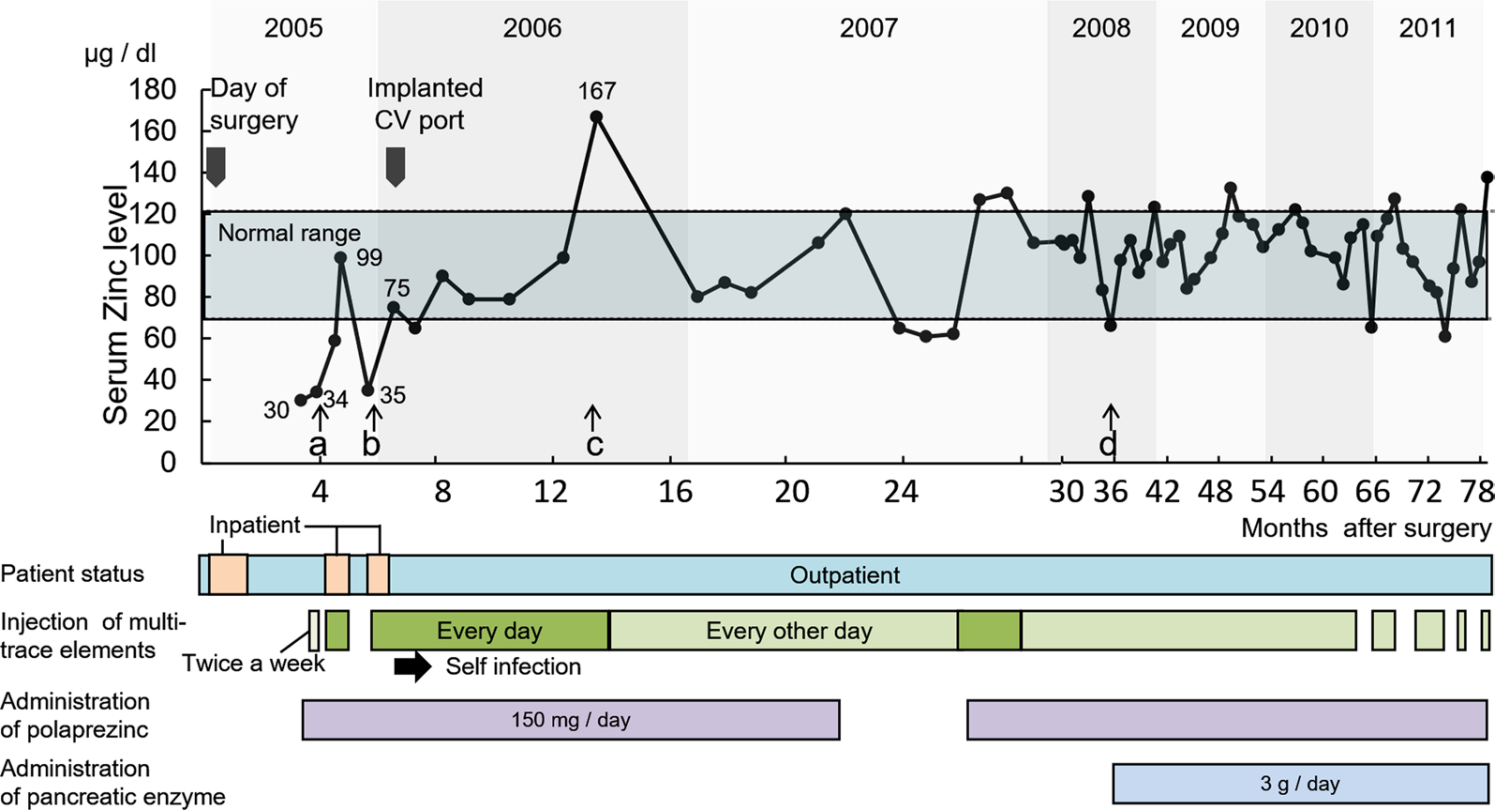
Transition of zinc supplementation and serum zinc level. Approximately 1 month after discharge following pancreaticoduodenectomy performed in July 2005, the patient experienced oral pain and dysgeusia caused by zinc deficiency (serum zinc level, 30 μg/dL). Oral zinc supplementation was inadequate, and dietary intake decreased; therefore, she was admitted to the hospital in November 2005 and administered intravenous supplementation of multi-trace elements (MTEs). (Arrow a), including zinc and high-calorie infusion. Serum zinc level increased to 99 μg/dL, and her food intake improved; hence, she was discharged 20 days after admission following intravenous zinc supplementation. However, after discharge from the hospital, intravenous zinc supplementation was discontinued, and she became hypozincemic again within a month (Arrow b); therefore, she was readmitted to the hospital at the end of December 2005. At the discharge in January 2006, a central venous port was indwelled, and home self-injection of MTEs was performed to maintain zinc levels

Initially, we orally administered 150 mg of Promac® granules 15% (polaprezinc, ZERIA Pharmaceutical Co., Ltd, Japan) per day (total zinc dose, 34 mg/day) to treat the zinc deficiency. However, due to the insufficient effectiveness of the replacement therapy, we additionally administered multi-trace elements (MTEs) for high-calorie infusions. Elemenmic® (Ajinomoto Co Inc, Japan) was administered intravenously, one ampule of which contained elemental iron (Fe) 35 μmol, manganese (Mn) 1 μmol, zinc (Zn) 60 μmol (= 4 mg), copper (Cu) 5 μmol, and iodine (I) 1 μmol; this was administered twice a week for 2 weeks as an outpatient treatment. However, the intravenous replacement therapy was similarly inadequate at this dosing interval and did not provide sufficient improvement in the serum copper and zinc values. The blood test results 4 months after PD were as follows: TP, 4.4 g/dL; ALB, 2.0 g/dL; Zn, 34 μg/dL; and Cu, 28 μg/dL; the patient required nutritional management during hospitalization with total parenteral nutrition (TPN) (Fig. 2 arrow a). An improvement was observed in the zinc level (99 μg/dL) and copper level (204 μg/dL) after 20 days of administering one ampule of Elemenmic® per day. Concurrently, her tongue pain and dysgeusia gradually improved. Because of a similar improvement in her nutritional status, she completed TPN (TP 5.1 g/dL, ALB 2.5 g/dL, Zn 99 μg/dL, Cu 204 μg/dL). A central venous catheter (CVC) inserted after admission was removed, and she was discharged 20 days after the second admission. On this occasion, intravenous zinc replacement therapy was discontinued.

One month after discharge, the serum zinc level decreased sharply, her oral pain increased again, and she had reduced food intake (Zn 35 μg/dL, Cu 44 μg/dL) (Fig. 2, arrow b). The patient was readmitted at the end of December 2005. To improve malnutrition, a CVC was re-inserted through the external jugular vein and injected with the MTE formulation and high-calorie infusions on consecutive days for 4 weeks (TP 5.9 g/dL, ALB 3.4 g/dL, Zn 75 μg/dL, Cu 47 μg/dL). In January 2006, since her oral pain and diet had improved, she was discharged from the hospital after receiving an implant of a central venous port, and she continued home self-injection of MTEs to maintain zinc levels. In February 2006, her taste function tended to improve, and in April 2006, the taste almost improved. When MTEs were self-injected daily, the serum zinc level gradually exceeded the normal range and reached 167 μg/dL in August 2006 (Fig. 2 arrow c); therefore, the administration was switched to every other day. Subsequently, we had to confirm the blood test data repeatedly to monitor serum zinc levels to ensure that they were within the normal range (Fig. 2). Despite continuing intravenous zinc replacement therapy, serum zinc levels decreased when additional oral zinc was discontinued in May 2007. After resuming the oral administration of Promac® due to the recurrence of dysgeusia, both the serum zinc level and dysgeusia improved. Since 2008, Pancreatin® (pancreatic enzyme, Mylan Co Inc, USA) 3 g/day had been administered alongside conventional zinc administration for this patient (Fig. 2, arrow d).

As of January 2012, she continued using MTEs intermittently while her serum zinc values were monitored; however, slight angular cheilitis was observed, and she exhibited no signs of glossitis and dysgeusia (Fig. 1c). Moreover, recurrence and metastasis of the primary tumor were not observed. She subsequently died of lung cancer in May 2020.

## Discussion and conclusions

There are few reports on postoperative taste loss in gastrointestinal cancer, and the involvement of zinc deficiency has not been investigated [16]. Since its introduction in 2002 at the Shizuoka Cancer Center Hospital, PD had been performed in approximately 45 patients per year until 2007; however, only the patient in this study experienced severe dysgeusia caused by zinc deficiency. In fact, dysgeusia and skin lesions caused by zinc deficiency following PD have not been assessed in quality-of-life studies [17–20]. The most common technique for PD consists of the en-bloc removal of the distal segment (antrum) of the stomach, the duodenum, the proximal part of the jejunum, the head of the pancreas, the common bile duct, and the gallbladder. Zinc is mainly absorbed in the duodenum and proximal jejunum, which are resected during PD, subsequently causing zinc deficiency. In the study by Yu et al. [13], one patient developed zinc deficiency with a serum zinc level of 32 μg/dL 15 months postoperatively. During hospitalization, zinc was supplemented intravenously, and the patient was discharged after an oral administration of pancreatic enzymes; another patient developed zinc deficiency with a serum zinc level of 29 μg/dL 4 years postoperatively. The patient required the continuous administration of zinc sulfate and pancreatic enzyme formulations [13]. In a study by Yazbeck et al., the patient developed zinc deficiency with a serum zinc level of 42 μg/dL 3 years postoperatively. The patient was discharged after an oral administration of 50 mg/day of zinc sulphate. Only in our case has oral zinc supplementation been inadequate, requiring continued intravenous zinc supplementation to maintain the normal serum zinc level. Although the details are unknown for other cases, the upper 15 cm part of the jejunum was surgically removed in this patient. Supposedly, conserved jejunum and ileum absorb zinc usually, and it is unclear why oral administration was inadequate in our case. Yu et al. and Yazbeck et al. proposed three mechanisms for zinc deficiency after PD [13, 21]: (1) impaired zinc absorption associated with jejunectomy; (2) insufficient protein absorption after PD, which occurs because zinc is transported in the serum via carrier proteins, namely ALB (57% of serum zinc), alpha-2-macroglobulin (40%), and amino acids, such as histidine and cysteine (< 3%) [13]; and (3) impaired fractional absorption of zinc due to pancreatic insufficiency, which is improved by exocrine pancreatic replacement [22]. Consequently, poor protein absorption may cause low zinc availability. Moreover, deficiencies in branched-chain amino acids and essential fatty acids [13] may contribute to the formation of skin lesions similar to those of acrodermatitis enteropathica observed in patients with zinc deficiency [23, 24]. From a therapeutic viewpoint, the clinical manifestation of zinc deficiency in PD might be improved not only by supplementation with zinc but also by the administration of pancreatic enzyme formulations and adequate intake of protein and essential fatty acids [13].

At the time of this study, the gastric ulcer healing agent Promac® 150 mg could be supplemented with 34 mg of zinc daily, while zinc replacement was an off-label use. In 2017, Nobelzin® tablet 50 mg (zinc acetate hydrate 167.84 mg, Nobelpharma Co. Ltd., Tokyo, Japan), which had been proven to be safe and effective for the long-term treatment of Wilson disease [25], was approved for the additional indication of hypozincemia in Japan [26]. Currently, for zinc replacement therapy, three tablets of Nobelzin® could be supplemented with at most 150 mg of zinc, which is more than four times as much zinc supplement as the standard dose of 150 mg of Promac® daily. The difference in bioavailability between zinc acetate hydrate and polaprezinc is unclear; however, in a study of zinc supplement for hemodialysis maintenance patients, 50 mg of zinc per day administered in the zinc acetate hydrate group was superior to 34 mg of zinc per day administered in the polaprezinc group in increasing and maintaining serum zinc levels [27]. It is unclear whether higher oral zinc supplementation with zinc acetate hydrate could replace intravenous zinc supplementation in our case. A limitation of this study is that the findings are not generalizable. Further research elucidating the probable causes of zinc deficiency after PD is required to improve the generalizability of our study findings.

Zinc deficiency after PD rarely occurs, and the mechanism has not been fully elucidated; the clinical oncologist community, including dentists responsible for the oral care of cancer patients, should be aware of the oral adverse events, such as dysgeusia, glossitis and oral pain, associated with zinc deficiency after cancer surgery, as well as that induced by chemotherapy or head and neck radiation therapy.

## Data Availability

All data generated or analyzed during this study are included in this published article.

## Abbreviations

PD: Pancreaticoduodenectomy
MTE: Multi-trace elements
CVC: Central venous catheter
TPN: Total parenteral nutrition
TP: Total protein
ALB: Albumin
